# Management of temperature in sepsis: a survey of current practice and opinion of uk intensive care consultants

**DOI:** 10.1186/2197-425X-3-S1-A217

**Published:** 2015-10-01

**Authors:** A Beverly, E Walters, M Carraretto

**Affiliations:** Royal Surrey County Hospital, Intensive Care Unit, Guildford, United Kingdom

## Intr

Sepsis is a leading cause of ICU admission, morbidity and mortality. Although guidelines exist to aid physicians in the early diagnosis and management of sepsis there exists no consensus for the optimal temperature for a patient with sepsis or the best means of achieving this.

## Methods

An anonymous on-line survey of ICU consultants regarding their current practice and attitudes to temperature control in treating hypo or hyperthermia in sepsis was conducted. Consultant practice regarding trigger and target temperatures, physical, invasive and pharmacological therapies was assessed. Surveys were distributed via the Critical Care Network nationally; response rate could not be calculated.

## Results

All respondents (N = 45) completed all 16 questions. The majority of respondents work in a general ICU, but in units with a wide range of bed numbers. 80% used cooling by physical means; 90% by pharmacological means. The temperatures at which respondents initiate cooling, or warming, and the level of concern, is shown in table 1. The most frequently used physical/invasive means were surface cooling (85%), intravascular cooling devices (50%) and cold water circulation (44%) whilst cold air circulation (20%) and cold gel pads (17%) were the least popular. Ten respondents do not use physical cooling, citing insufficient evidence (70%), lack of national (50%) or departmental (50%) guidelines and lack of equipment (13%) as reasons. Preferred agents for pharmacological temperature reduction were regular paracetamol (82%), PRN paracetamol (65%), regular NSAIDS (4%) and PRN NSAIDS (12%). Six respondents do not employ pharmacological cooling, citing insufficient evidence base (100%) and lack of national guidance (50%) as reasons. 18% of respondents had departmental guidelines for temperature management in sepsis.

**Table 1:** Clinician response to temperatures in sepsis

Temperature-->	<34 C	34-35 C	35-36 C	36-37 C	37-38 C	38-39 C	39-40 C	40-41 C	>41 C
I would use pharmacological cooling					12.3%	66.4%	86.1%	88.6%	90.7%
I would use physical/invasive cooling					6.3%	18.9%	56.8%	77.8%	80%
I would use physical/invasive warming	83.7%	79.5%	56.8%	2.3%					
This temperature causes slight concern	4.8%	34.2%	52.5%	7.3%	14.6%	65.9%	43.9%	5.0%	0%
This temperature causes severe concern	95.2%	63.4%	7.5%			9.8%	53.7%	95.0%	100%

## Conclusions

While a raised temperature in sepsis is likely to be beneficial, too high a temperature is harmful, but it is not clear even now if there is an optimum temperature, or whether physical or pharmacological cooling is beneficial or harmful [1]. There was considerable spread in this survey around the trigger for manipulating temperature in sepsis, but correlates with the level of perceived concern at that temperature. Of those who don't manipulate temperature, lack of evidence, and lack of guidelines are the commonest causes. This survey highlights the lack of consensus on the optimum target temperature, and the need for further work and guidance.

## References

[1] Young PJ, Saxena M (2014). Fever management in intensive care patients with infections. Critical care.

